# Assessment of the Fibrosis Score and the Child-Turcotte-Pugh (CTP) Score in Patients With Chronic Liver Disease in India

**DOI:** 10.7759/cureus.74728

**Published:** 2024-11-29

**Authors:** Madhavi Sarkari, Smita Chaudhary, Bechan Kumar Gautam

**Affiliations:** 1 Department of Medicine, Baba Raghav Das Medical College, Gorakhpur, Gorakhpur, IND

**Keywords:** ascites, child-pugh-turcotte score, chronic liver disease, cirrhosis, fibrosis score

## Abstract

Objective: This study aimed to evaluate the severity of liver fibrosis in chronic liver disease patients using aspartate aminotransferase-to-platelet ratio index (APRI), fibrosis-4 (FIB-4), FibroScan, and the Child-Turcotte-Pugh (CTP) score. It emphasized assessing fibrosis progression toward cirrhosis (F4 stage) and exploring the correlation between non-invasive markers and the CTP score for liver function and prognosis.

Methodology: This observational cross-sectional study was conducted over one calendar year in the Department of Medicine at Baba Raghav Das (BRD) Medical College, Gorakhpur, India. A total of 200 patients with chronic liver disease were selected. Fibrosis scores were calculated using FibroScan, APRI, and FIB-4, while the modified CTP score was determined for each participant. Pearson’s correlation was used to assess relationships between variables, while logistic regression evaluated the association of non-invasive methods (APRI, FIB-4, FibroScan) with severe fibrosis (F4). Odds ratios (ORs), sensitivity, specificity, and AUC were calculated, and ROC curves visualized their discriminative ability. Statistical significance was defined as p < 0.05.

Results: The study revealed a predominance of advanced fibrosis (F4) in males (82.5%) and patients with ethanol-induced liver disease (84.6%). FIB-4 had the strongest predictive value for advanced fibrosis with an OR of 3.8 (95% CI: 3.0-4.5) and AUC of 0.743, followed by APRI with an OR of 2.5 (95% CI: 1.9-3.1) and AUC of 0.757. CTP showed the highest sensitivity (95.45%) but a lower AUC (0.697), indicating its clinical value in correlating fibrosis severity with liver dysfunction. Hemoglobin, platelets, and INR showed no significant correlation with fibrosis, while total bilirubin was elevated in advanced CTP classes. A moderate positive correlation (r = 0.481, p < 0.001) was observed between fibrosis scores and CTP, linking fibrosis severity with liver dysfunction. These findings emphasize FIB-4’s superior predictive accuracy, while APRI and CTP remain valuable complementary tools for liver disease prognosis.

Conclusion: In conclusion, FIB-4 is the most accurate for staging advanced fibrosis, while APRI excels in initial screening due to its higher sensitivity. FibroScan effectively assesses direct fibrosis, and the CTP score adds prognostic value, making these methods complementary for managing chronic liver diseases.

## Introduction

Cirrhosis, the final stage of many chronic liver diseases (CLD), is marked by extensive hepatic fibrosis and the formation of nodules that disrupt the normal liver structure [1]. It is a leading cause of death in the United States, particularly impacting individuals in their most productive years. Globally, the mortality rate from CLD has risen significantly, with Asia and Africa experiencing the highest increases [2]. In recent years, cirrhosis has become a major global health concern, and earlier projections had estimated it would rank as the 12th leading cause of death by 2020 [3]. Alcohol remains the primary cause of CLD in regions such as Asia, including India [4].

The prognosis for cirrhosis depends on its clinical stage and the presence of comorbidities. Individuals with compensated cirrhosis have a median survival of 9-12 years, whereas those with decompensated cirrhosis have a much shorter median survival of approximately two years [5]. Diagnosis typically relies on clinical symptoms, biochemical tests, and hepatic imaging, although abnormalities often become apparent only in moderate to advanced stages of the disease. Blood test results have been integrated into various predictive models for advanced liver disease, and scoring systems such as the modified Child-Pugh classification are commonly used to evaluate prognosis. This classification assigns a score ranging from 5 to 15, with scores of 5-6 indicating compensated cirrhosis (Child-Pugh Class A), 7-9 indicating Class B, and 10-15 indicating Class C [6].

Despite the liver’s remarkable regenerative capacity, chronic damage results in progressive hepatic fibrosis, which may regress if the underlying cause is eliminated [7]. The severity of liver fibrosis in CLD patients is a critical predictor of liver-related complications and mortality [8]. Consequently, assessing fibrosis is essential for determining treatment strategies, monitoring disease progression, and evaluating therapeutic success [9]. In clinical research, fibrosis has also served as a key surrogate endpoint, expediting the approval of effective drug therapies. Although liver biopsy remains the gold standard for fibrosis assessment, its invasive nature and associated risks limit its use [10]. Non-invasive alternatives, such as FibroScan (transient elastography), the aspartate aminotransferase to platelet ratio index (APRI), and the Fibrosis-4 (FIB-4) index, have proven effective in detecting or ruling out advanced fibrosis. These methods allow a significant proportion of patients to avoid biopsy. FibroScan, in particular, assesses disease severity by measuring the mechanical properties of the fibrotic liver [11-13].

Meanwhile, the aspartate aminotransferase-to-platelet ratio index (APRI), a non-invasive biomarker initially developed for patients with chronic hepatitis B and C, has also been applied to predict liver-related outcomes in conditions such as alcoholic liver disease and chronic hepatitis C virus infection [6-8]. It is inexpensive and simple to calculate and provides reliable predictions of fibrosis and cirrhosis, with high negative and positive predictive values. Despite some controversies over its clinical utility, the APRI remains a widely used tool, especially in outpatient settings, for identifying patients who require additional care [14,15].

In this study, we aimed to assess the severity of liver fibrosis in chronic liver disease patients using four evaluation methods: APRI, FIB-4, FibroScan (transient elastography), and the Child-Turcotte-Pugh (CTP) score. The analysis focused on evaluating the progression of fibrosis, particularly toward cirrhosis (F4 stage), while examining the correlation between non-invasive markers and the CTP score as a measure of liver function and prognosis.

## Materials and methods

Study design

This observational, cross-sectional study was conducted from May 2023 to June 2024 in the Department of Medicine at Baba Raghav Das (BRD) Medical College, Gorakhpur, India.

Setting

The study was conducted at BRD Medical College, Gorakhpur, a prominent tertiary care teaching hospital in eastern Uttar Pradesh, India. As a major regional referral center, the institution offers comprehensive diagnostic and therapeutic services for various medical conditions, including chronic liver diseases. The hospital has 1,000 inpatient beds and provides care to approximately 250,000 outpatients and 30,000 inpatients annually. The Department of Medicine, where the study was carried out, is staffed by specialists in gastroenterology and hepatology and is well-equipped with state-of-the-art diagnostic tools, including transient elastography (FibroScan) and ultrasound-guided procedures. This well-resourced setting allowed for the detailed evaluation of liver disease severity in the study population.

Study population

The study included 200 patients with chronic liver disease, defined as persistent inflammation and fibrosis of the liver lasting more than six months. Participants were recruited according to strict inclusion and exclusion criteria. Eligible patients were 18 years or older and had a suspected diagnosis of CLD based on clinical, biochemical, and imaging findings. Exclusion criteria included acute liver failure, hepatocellular carcinoma, malignancies other than CLD, and systemic illnesses that could independently impair liver function. All participants provided written informed consent before their enrollment.

Sample size collection

The sample size of 200 was determined using a statistical formula based on a prevalence rate of 18.3% for advanced fibrosis reported by Mondal et al. [16], ensuring statistical reliability with a 95% confidence level and a 5.5% margin of error. This sample size aligns with the study objectives by providing sufficient representation of fibrosis severity groups (≤ F3 and F4) and enabling robust subgroup analyses based on CTP classes (A, B, and C). By slightly exceeding the minimum required sample size of 189, the study ensures reliable sensitivity, specificity, and correlation analyses while accounting for variability in fibrosis staging. Additionally, the sample reflects the large patient population treated at BRD Medical College, enhancing the clinical applicability of the findings.

Data collection

Comprehensive demographic and clinical data were systematically recorded for each participant using a predesigned case record form. All participants underwent routine laboratory investigations, which included a complete blood count, liver function tests (LFT), renal function tests (RFT), and urinalysis. Coagulation parameters, such as prothrombin time, international normalized ratio (INR), and activated partial thromboplastin time (APTT), were also assessed. INR, in particular, was used as an essential indicator of liver synthetic function and coagulation status. In addition, serological tests for hepatitis B surface antigen (HBsAg) and hepatitis C virus (HCV) antibodies were performed.

For patients presenting with ascites, the severity was graded as mild (detectable only by ultrasound), moderate (detectable on clinical examination), or severe (tense ascites causing abdominal distension). Ascitic fluid analysis was performed to determine the serum ascitic albumin gradient (SAAG) ratio and to rule out infections or malignancies through cytological and microbiological assessments. Abdominal ultrasound was used to evaluate liver echotexture, spleen size, portal vein diameter, and the presence and severity of ascites. Additionally, transient elastography was employed to quantify liver stiffness and assess hepatic steatosis using the controlled attenuation parameter (CAP) score.

Assessment of fibrosis

Liver fibrosis was evaluated non-invasively using transient elastography (FibroScan), which measured liver stiffness. Fibrosis severity was categorized into stages ranging from F0 (no fibrosis) to F4 (cirrhosis) based on kilopascal (kPa) readings. Non-invasive biochemical indices, including the APRI and FIB-4 scores, were also calculated using established formulas. These biochemical scores were compared with FibroScan measurements to enhance the accuracy of fibrosis staging.

CTP score

The CTP score was calculated for all participants to assess the degree of liver dysfunction. The score included five clinical and biochemical parameters: serum bilirubin levels, serum albumin levels, INR, the severity of ascites (graded clinically or through ultrasound), and hepatic encephalopathy. Based on the total score, patients were classified into three categories: Class A (5-6 points), indicative of well-compensated liver disease; Class B (7-9 points), indicative of significant functional impairment; and Class C (10-15 points), indicative of decompensated liver disease.

Statistical analysis

Statistical analysis was conducted by using Statistical Product and Service Solutions (SPSS, version 23.0; IBM SPSS Statistics for Windows, Armonk, NY). Continuous variables were expressed as mean ± standard deviation (SD), while categorical variables were reported as frequencies and percentages. Pearson’s correlation coefficient was used for correlation. In addition, logistic regression analysis was performed to evaluate the association between non-invasive methods (APRI, FIB-4, and FibroScan) and the likelihood of severe fibrosis (F4). ORs with 95% confidence intervals (CIs) were calculated to quantify the predictive power of each method. Sensitivity, specificity, and area under the curve (AUC) were calculated for each method using the probabilities derived from logistic regression. Receiver operating characteristic (ROC) curves were plotted to visualize the discriminative ability of these non-invasive tools, with a p-value of less than 0.05 considered statistically significant.

## Results

The average age varied from 45.58 ± 19.55 years (F3) to 52 ± 16.81 years (F2), resulting in an overall mean of 47.7 ± 13.93 years. Of the total, 155 (77.5%) were males, with the majority in F4, totaling 128 (82.5%). Ethanol-induced liver disease was observed in 136 (68%) individuals, primarily classified as F4 (98, 72%), whereas metabolic dysfunction-associated fatty liver disease (MAFLD) affected 44 (22%) individuals, predominantly in F4 (33, 75%). Only F4 showed HBsAg positive (21, 10.5%) and HCV (4, 2%) cases, while cryptogenic and autoimmune hepatitis were infrequent (2, 1% each). In patients without ascites (137, 68.5%), the majority were classified as F4 (106, 77.3%), but minor ascites (52, 26%) and moderate/severe ascites (11, 5.5%) were also primarily observed in F4. Hepatic encephalopathy was not present in 145 (72.2%) instances, while weak-to-moderate cases were observed in 48 (24%) and severe cases in seven (3.5%), primarily in F4. The data indicate an increased prevalence of ethanol-induced illness, MAFLD, ascites, and encephalopathy in advanced fibrosis (F4) (Table 1).

**Table 1:** Distribution of clinical and demographic parameters across fibrosis stages MAFLD: metabolic-associated fatty liver disease, HBsAg: hepatitis B surface antigen, HCV: hepatitis C virus

Parameters	Fibrosis Stages				Total number of patients
	F0-F1	F2	F3	F4	
Age (in years)	46.65 ± 16	52 ± 16.81	45.58 ± 19.55	47.84 ± 13.04	47.7 ± 13.93
Gender/Male (N,%)	12 (7.7%)	7 (4.5%)	8 (5.1%)	128 (82.5%)	155 (77.5%)
Ethanol-induced disease (N,%)	3 (2.2%)	4 (2.9%)	15 (11.02%)	98 (72%)	136 (68%)
MAFLD (N,%)	1 (2.27%)	2 (4.54%)	3 (6.8%)	33 (75%)	44 (22%)
HBSAg (N,%)	0 (0%)	0 (0%)	0 (0%)	21 (100%)	21 (10.5%)
HCV (N, %)	0 (0%)	0 (0%)	0 (0%)	4 (100%)	4 (2%)
Cryptogenic (N,%)	0 (0%)	(0%)	1 (50%)	1 (50%)	2 (1%)
Autoimmune hepatitis (N,%)	0 (0%)	0 (0%)	0 (0%)	2 (100%)	2 (1%)
Ascites severity (N,%)					
None	16 (11.6%)	7 (5.1%)	8 (5.83%)	106 (77.3%)	137 (68.5%)
Slight	2 (3.84%)	1 (1.92%)	4 (7.6%)	45 (86.5%)	52 (26%)
Moderate/ Severe	0 (0%)	1 (9.09%)	0 (0%)	10 (90.9%)	11 (5.5%)
Hepatic Encephalopathy (N,%)					
None	15 (10.5%)	8 (5.5%)	9 (6.2%)	113 (77.9%)	145 (72.2%)
Slight/Moderate (Grades I-II)	3 (6.2%)	1 (2%)	2 (4.1%)	42 (87.5%)	48 (24%)
Moderate/Severe (Grades III-IV)	0 (0%)	0 (0%)	1 (14.2%)	6 (85.7%)	7 (3.5%)

The study investigated the correlation between fibrosis and essential laboratory values in CTP Class B and C patients. The two groups had similar levels of hemoglobin (9.15 ± 2.27 g/dL in Class B and 9.53 ± 2.19 g/dL in Class C), and there was no link between them and fibrosis (r = 0.007, p = 0.92). Class B and Class C had platelet counts of 44.94 ± 35.98 x 10⁹/L and 45.12 ± 35.79 x 10⁹/L, respectively, with no significant correlation (r = -0.08, p = 0.29). Class C exhibited a significant increase in total bilirubin levels (13.61 ± 9.90 mg/dL) in comparison to Class B (3.19 ± 3.01 mg/dL); however, the relationship was negligible (r = -0.01, p = 0.79). The serum albumin levels in Class C (2.60 ± 0.47 g/dL) were slightly lower than those in Class B (2.79 ± 0.53 g/dL), but there was no significant correlation (r = -0.003, p = 0.96). The INR was higher in Class C (1.79 ± 0.51) than in Class B (1.55 ± 0.46), but the correlation was not significant (r = 0.13, p = 0.09). The results suggest that there are no significant relationships between fibrosis and these parameters in the population under investigation (Table 2).

**Table 2:** Comparison of clinical parameters between CTP Classes B and C with correlation to fibrosis

Parameter	CTP Class B (Mean ± SD)	CTP Class C (Mean ± SD)	Correlation with Fibrosis (r)	p-value
Hemoglobin (g/dL)	9.15 ± 2.27	9.53 ± 2.19	0.007	0.92
Platelets (x10⁹/L)	44.94 ± 35.98	45.12 ± 35.79	-0.08	0.29
Total Bilirubin (mg/dL)	3.19 ± 3.01	13.61 ± 9.90	-0.01	0.79
Serum Albumin (g/dL)	2.79 ± 0.53	2.60 ± 0.47	-0.003	0.96
INR	1.55 ± 0.46	1.79 ± 0.51	0.13	0.09

**Figure 1:**
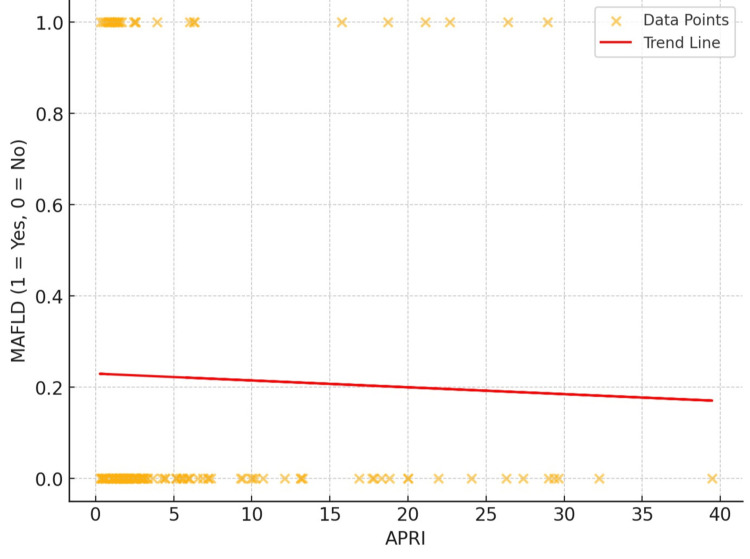
Correlation between APRI and MAFLD APRI: aspartate aminotransferase-to-platelet ratio index; MAFLD: metabolic dysfunction-associated fatty liver disease

The results indicate a modest negative association between APRI and MAFLD, with a correlation coefficient (r) of -0.113 and a p-value of 0.118, suggesting that the association lacks statistical significance. Although APRI readings indicate a marginal reduction in MAFLD prevalence, this correlation lacks predictive power and clinical relevance. The graph suggests that APRI is not a reliable tool for assessing the fibrosis stage in relation to MAFLD (Figure 1).

**Figure 2:**
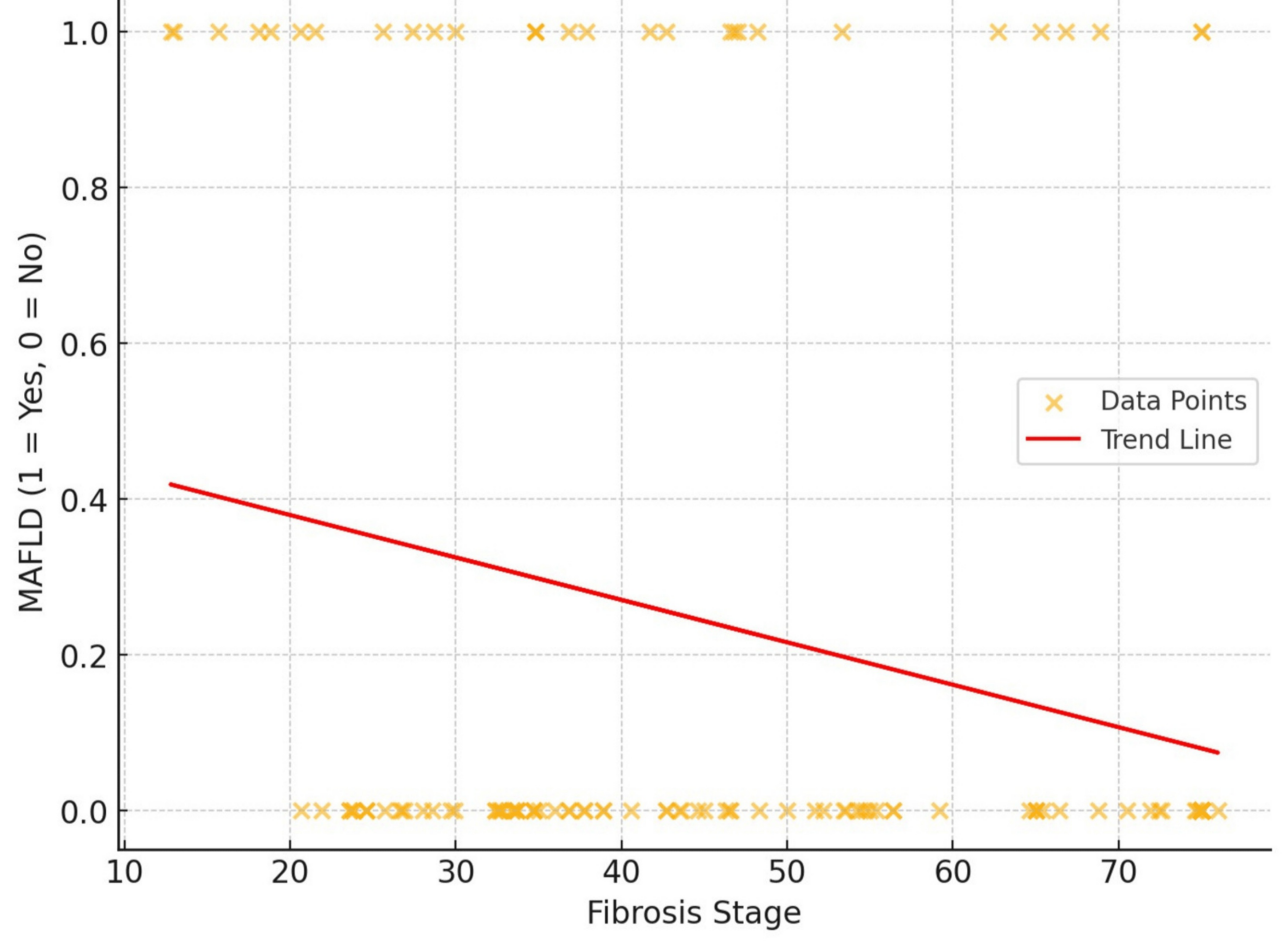
Correlation between fibrosis stage and MAFLD MAFLD: metabolic dysfunction-associated fatty liver disease

The results demonstrate a negative association between gibrosis stage and the occurrence of MAFLD, indicating potential consequences for illness prognosis. The trend line indicates a decrease in MAFLD prevalence from roughly 0.4 at lower fibrosis stages (around 10) to below 0.2 at higher fibrosis stages (around 70). The correlation coefficient (r) of -0.259 indicates a moderate negative correlation, and a p-value of 0.0027 demonstrates its statistical significance. This indicates that, as fibrosis advances to later stages, the probability of concurrent MAFLD diminishes. This tendency highlights the potential differences in the pathophysiological course of advanced fibrosis, implying that metabolic-associated variables such as MAFLD may have less impact on the prognosis of liver disease in elevated fibrosis stages (Figure 2).

**Figure 3:**
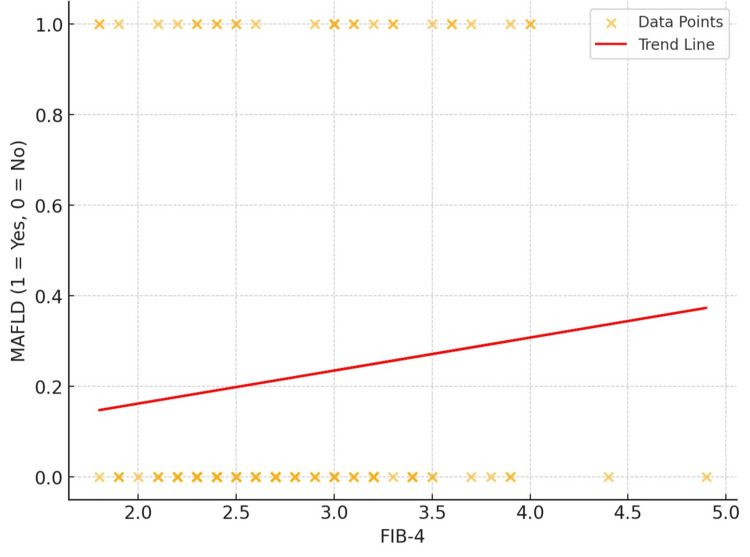
Correlation between FIB-4 and MAFLD FIB-4: fibrosis-4; MAFLD: metabolic dysfunction-associated fatty liver disease

The graph shows a slight positive association between FIB-4 and the occurrence of MAFLD, with a correlation coefficient (r) of 0.099, indicating a marginal increase in MAFLD prevalence as FIB-4 values rise. The association is statistically insignificant, as indicated by a p-value of 0.259. This suggests that, while there is a minor positive connection, it may not be clinically relevant in anticipating MAFLD based only on FIB4 (Figure 3).

**Figure 4:**
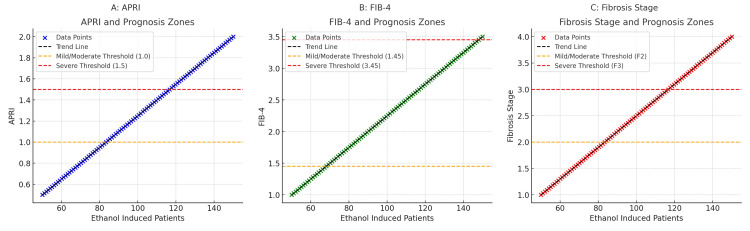
Comparison of APRI, FIB-4, and fibrosis stages across prognostic zones in ethanol-induced patients APRI: aspartate aminotransferase-to-platelet ratio index; FIB-4: fibrosis-4

The results reveal a positive connection between ethanol exposure and APRI, FIB-4, and fibrosis stage. The APRI values consistently rise, surpassing the mild/moderate threshold of 1.0 and nearing the severe threshold of 1.5, signifying a decline in liver function due to increased ethanol consumption. APRI may not be a precise technique for staging fibrosis in ethanol-induced cases despite the fact that it clearly shows liver failure and inflammation. FIB-4 is a solid indicator of fibrosis severity because of its continually increasing trajectory, which exceeds the mild/moderate barrier of 1.45 and approaches the severe threshold of 3.45. The fibrosis stage is a direct measure of fibrosis that ranges from mild/moderate fibrosis (F2) to severe fibrosis (F3), offering the most accurate assessment of the disease's progression. Findings show that FIB-4 and the fibrosis stage are more precise ways to measure fibrosis severity than APRI. These findings underscore that increased alcohol use exacerbates the condition and leads to progressive liver impairment (Figure 4).

Table 3 shows that CTP class, APRI score, and FIB-4 score are strong predictors of the fibrosis stage, with varying degrees of association across severity levels. In comparison to the low fibrosis group, a higher CTP class increases the likelihood of being categorized as moderate fibrosis by a factor of 2.0 (95% CI: 1.5-2.5, statistically significant). Each unit increase in the APRI score increases the chances by 1.8 times (95% CI: 1.2-2.3, statistically significant), while the odds for FIB-4 increase by 2.2 times (95% CI: 1.8-2.8, statistically significant). The OR for high versus reference (low) fibrosis stage are statistically significant for all three parameters. An increased CTP class increases the likelihood of belonging to the high fibrosis group by 3.2 times compared to the low fibrosis group (95% CI: 2.5-4.0, statistically significant). Furthermore, each unit increment in the APRI score increases the risk by 2.5 times (95% CI: 1.9-3.1, statistically significant), but each unit increment in FIB-4 increases the odds by 3.8 times (95% CI: 3.0-4.5, statistically significant), demonstrating the strongest relationship. The data show a progressive rise in OR with increased fibrosis severity, emphasizing the importance of these measures in assessing disease progression. FIB-4 has the highest OR of the predictors, making it the most sensitive and accurate biomarker for predicting advanced fibrosis. Although the CTP class and APRI score are relevant, FIB-4's improved predictive potential highlights its clinical relevance for the prognosis of advanced liver disease (Table 3).

**Table 3:** Logistic regression odds for fibrosis stages among all etiologies

Predictor	Low	Moderate	High
CTP class	1.2 (0.9 to 1.5)	2.0 (1.5 to 2.5)	3.2 (2.5 to 4)
APRI score	1.1 (0.8 to 1.4)	1.8 (1.2 to 2.3)	2.5 (1.9 to 3.1)
FIB-4 score	1.5 (1.2 to 1.8)	2.2 (1.8 to 2.8)	3.8 (3 to 4.5)

**Figure 5:**
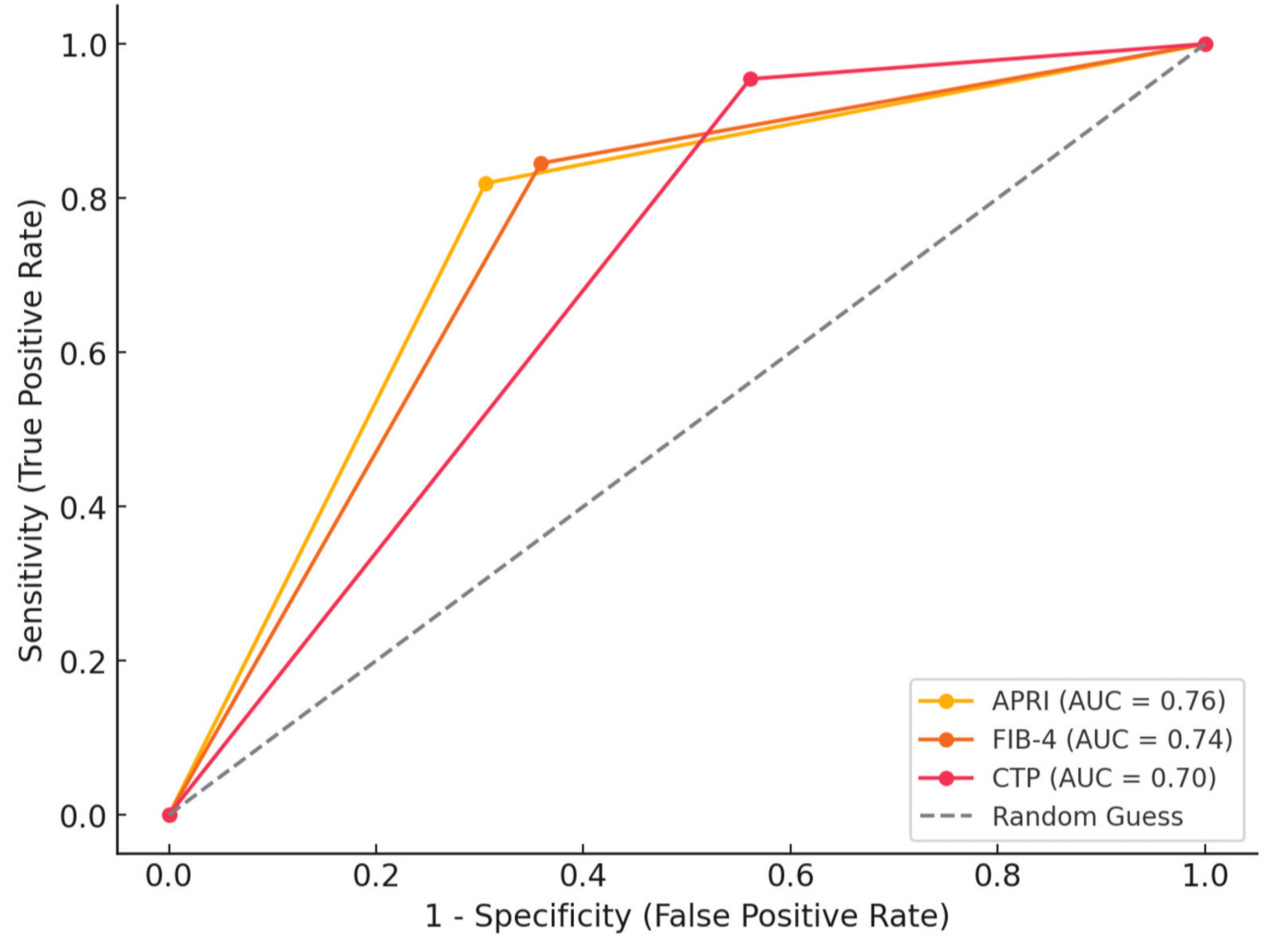
ROC curve for APRI, FIB-4, and CTP APRI: aspartate aminotransferase-to-platelet ratio index; FIB-4: fibrosis-4; CTP: Child-Turcotte-Pugh; ROC: receiver operating characteristic

Figure 5 depicts the diagnostic effectiveness of APRI, FIB-4, and CTP in assessing stages of liver fibrosis. APRI exhibited a sensitivity of 81.94%, specificity of 30.56%, and the highest AUC of 0.7569, demonstrating reasonable reliability in identifying fibrosis phases. The FIB-4 had a sensitivity of 84.52%, a specificity of 35.89%, and an AUC of 0.74309, indicating equal diagnostic accuracy to APRI. CTP had the highest sensitivity at 95.45% and specificity at 56.10%, although its AUC of 0.69678 was slightly lower. Although APRI and FIB-4 had higher overall accuracy, CTP provided additional clinical value due to its high sensitivity and ability to correlate with advanced fibrosis stages.

## Discussion

This study assessed the reliability of non-invasive methods - CTP score, FibroScan, APRI, and FIB-4 - in predicting fibrosis progression in chronic liver disease, particularly in advanced stages such as cirrhosis (F4). The findings provide significant insights into the therapeutic effectiveness of these approaches, aligning with and, at times, diverging from prior international and regional research.

The FIB-4 index, with an OR of 3.8 (95% CI: 3.0-4.5) and an AUC- ROC of 0.74, proved highly accurate in diagnosing high-risk fibrosis, particularly in advanced stages (F3-F4). These results are consistent with studies by Sterling et al. [17] and Shah et al. [18], which highlighted FIB-4 as a reliable method for detecting advanced fibrosis in chronic hepatitis C and non-alcoholic fatty liver disease (NAFLD). Similarly, Bertot et al. [19] reported an AUC of 0.80 (95% CI: 0.75-0.85) for FIB-4 in predicting advanced fibrosis. However, this study found weaker associations between FIB-4 and metabolic liver diseases, such as MAFLD, than Shah et al. [18] and Angulo et al. [20]. These discrepancies highlight the need for region-specific research to validate FIB-4’s diagnostic consistency.

The APRI index, with an AUC of 0.76 and an OR of 2.5 (95% CI: 1.9-3.1), demonstrated notable sensitivity for diagnosing early-stage fibrosis (F2-F3). This result aligns with findings by Wai et al. [11] and Lin et al. [21], who reported APRI as effective for detecting severe fibrosis, particularly in resource-limited settings. However, its accuracy diminished in staging advanced fibrosis, particularly in cases of ethanol-induced liver disease and MAFLD. Bertot et al. [19] noted similar variability, with an AUC of 0.73 (95% CI: 0.68-0.78) for APRI in NAFLD patients. Contrary to Sebastiani et al. [22], who deemed APRI reliable for advanced fibrosis staging, this study identified its limitations later, suggesting the complementary use of FIB-4 for improved accuracy. While APRI demonstrates significant sensitivity for early fibrosis detection, its limitations in advanced stages align with findings by Wai et al. [11] and Lin et al. [21].

FibroScan emerged as a dependable, non-invasive tool for assessing hepatic stiffness, as demonstrated by Castera et al. [23] and Wong et al. [24]. Bertot et al. [19] found that FibroScan outperformed APRI and FIB-4 in detecting advanced fibrosis, with an AUC of 0.85 (95% CI: 0.81-0.89). While this study confirmed FibroScan’s effectiveness for advanced fibrosis diagnosis, it provided limited quantitative data, reinforcing its established preference in well-funded clinical settings.

The CTP score showed limited predictive efficacy for fibrosis staging compared to FIB-4. This study corroborates the findings by Friedrich-Rust et al. [25], who demonstrated comparable diagnostic accuracy using imaging techniques such as FibroScan and non-invasive indicators such as FibroTest and ELF. However, unlike Friedrich-Rust et al. [25], who emphasized complex diagnostic methods, this study highlighted the utility of clinical indicators like FIB-4 and APRI, especially in resource-constrained settings. Combining these methods in various clinical contexts underscores the balance between cost-effectiveness and diagnostic precision.

Additional evidence from Mózes et al. [26] and McPherson et al. [27] supports the clinical efficacy of non-invasive scoring systems, showing that FIB-4 and APRI effectively detect advanced fibrosis in NAFLD patients. Variations in diagnostic performance across populations, particularly in metabolic liver diseases, emphasize the need for further research to develop population-specific risk scores and validate these tools for broader application.

Strengths and limitations of the study

This study provides valuable insights into non-invasive liver fibrosis assessment by comparing the reliability of APRI, FIB-4, and FibroScan against the CTP score. While earlier research often focused on these indicators individually, this study offers a comprehensive comparison, highlighting their strengths and limitations in predicting fibrosis progression, particularly in advanced stages such as cirrhosis (F4). By establishing FIB-4 as a reliable predictor of advanced fibrosis and confirming APRI’s sensitivity for early detection, this work helps clarify inconsistencies in previous studies. Additionally, it addresses gaps in research relevant to resource-limited settings, where cost-effective tools such as APRI and FIB-4 are crucial for patient care. The study also explores emerging challenges, such as the weaker correlations between MAFLD and non-invasive markers, shedding light on less-studied aspects of chronic liver disease.

However, our study has certain limitations. The findings may have restricted generalizability due to the specific cohort studied, potentially limiting applicability to other demographics or causes of liver disease. The FibroScan analysis, while acknowledged, lacks a detailed quantitative comparison with different methods, which could have strengthened its evaluation. Furthermore, the cross-sectional design restricts the ability to assess fibrosis progression over time. Variability among subgroups, as evidenced by weaker relationships in MAFLD cases, also poses challenges to broader generalizability. Despite these limitations, this study makes a significant contribution to the field by providing a foundation for future research aimed at addressing these challenges and enhancing the effectiveness of non-invasive diagnostic tools.

## Conclusions

In conclusion, the results show that FIB-4 is the most accurate tool for staging fibrosis and has a high accuracy rate for predicting advanced fibrosis. However, APRI is better for initial screening and diagnosing patients who are likely to progress because it has a higher sensitivity rate. FibroScan is a solid option for assessing direct fibrosis, although the CTP score provides valuable information about overall liver function and prognosis. These strategies complement one another, making them more effective for the early identification, staging, and treatment of chronic liver diseases. Future research should focus on validating these findings across multiple populations and incorporating these tools into predictive models to improve diagnostic precision and allow for more personalized treatment methods.
